# Direct and indirect effects of physical exercise on life satisfaction and quality of life in Chinese adolescents during the COVID-19 pandemic

**DOI:** 10.3389/fpubh.2026.1692392

**Published:** 2026-01-22

**Authors:** Liqiang Dou, Mi Ma, Wenbo Qi, Honghong Ren, Guoqing Zhao, Ping Sun

**Affiliations:** 1 Department of Psychiatry, Qingdao University Medical College, Qingdao, China; 2 Department of Psychiatry, Pingdu Mental Health Center, Qingdao, China; 3 Department of Psychiatry, Qingdao Mental Health Center, Shandong, China; 4 Department of Psychology, Shandong Provincial Hospital Affiliated to Shandong First Medical University, Jinan, Shandong, China

**Keywords:** adolescents, anxious symptoms, depressive symptoms, life satisfaction, physical exercise, quality of life

## Abstract

**Background:**

The COVID-19 pandemic exacerbated mental health challenges among adolescents, yet the protective role of regular physical exercise (PE) in enhancing life satisfaction (LS) and quality of life (QOL) remains underexplored in this population. This study aimed to investigate the direct and indirect effects of PE on LS and QOL among Chinese adolescents during the pandemic, with a focus on its mediating pathways through alleviating depressive and anxious symptoms.

**Methods:**

Adolescents aged 12 to 17 seeking for psychological services at Shandong Provincial Hospital were recruited between June 2021 and May 2022. Using the Patient Health Questionnaire-9 (PHQ-9), Generalized Anxiety Disorder-7 (GAD-7), 6-items QOL questionnaire (QOL-6), and Adolescent Students’ Life Satisfaction Scale (ASLSS), we assessed depressive symptoms, anxious symptoms, QOL, and LS.

**Results:**

A total of 392 adolescents (133 regular physical exercisers (PEs) vs. 259 non-regular physical exercisers (NPEs)) were included. Significant differences were observed in educational level, residence, general health status, and regular diet between PE and NPEs. A one-way ANOVA showed that PEs had significantly lower scores on GAD-7 and PHQ-9, higher QOL-6 scores, and greater LS across all six ASLSS dimensions compared to NPEs. The mediation analysis showed regular exercise indirect positive effects on QOL-6 and ASLSS 6 dimensions through the alleviation of anxious and depressive symptoms. Stepwise regression confirmed the significant positive effect of exercise on QOL, as well as LS with friendship, academic performance, and autonomy.

**Conclusion:**

PE alleviates emotional distress and enhances subjective well-being in adolescents through dual pathways, underscoring its value as a low-cost intervention during public health crises. Findings advocate integrating structured physical activity into school-based mental health programs to foster resilience. Longitudinal studies are needed to validate causal mechanisms and optimize exercise protocols for diverse adolescent populations.

## Introduction

Physical exercise (PE) is a well-established promoter of physical and mental health across populations (1). For individuals with psychiatric conditions, PE offers significant biological and psychological benefits, including enhanced metabolism, neuroprotection, and reduced psychopathological symptoms (2–5). Interventions for severe mental illnesses suggest PE may not only alleviate symptoms but also inhibit disease progression (6). The mechanisms underlying these benefits are best understood through a biopsychosocial lens: PE modulates neuroendocrine responses (e.g., reducing cortisol, releasing endorphins), enhances psychological resources (e.g., self-efficacy, self-esteem), and strengthens social connections (7–9). Consequently, PE is linked to improved mood, reduced anxiety and depression, and a higher quality of life (QOL).

The role of PE is particularly critical during adolescence, a period of profound physiological and psychological development. Evidence indicates that PE and sports participation contribute to adolescents’ cognitive health, academic performance, perceived social competence, and psychological well-being (10–13). Despite these multifaceted benefits, modern lifestyle shifts pose significant barriers to PE engagement among adolescents. A pervasive increase in screen time and sedentary behavior, notably exacerbated by the disruption of routines during the COVID-19 pandemic, is associated with declining PE participation and poorer mental health outcomes (14–17).

The unique context of Chinese adolescents during the pandemic necessitates focused investigation. China’s high-pressure academic environment, combined with some of the world’s most stringent and prolonged lockdown measures, created a perfect storm that uniquely impacted adolescent well-being. The transition to distance learning, severe restrictions on social interaction, and disruption of daily structure likely intensified screen dependence while simultaneously stripping away key outlets for stress relief and social development (18–21). This context makes the Chinese adolescent population, especially those already seeking psychological services, a critical group for examining PE’s protective potential. Within this crisis, PE’s role as a biopsychosocial buffer against emotional distress demands urgent exploration.

However, significant gaps remain. While the general benefits of PE are documented, research has yet to explore how the specific trends of declining PE and rising screen time affect life satisfaction (LS) in this high-risk context. Crucially, no studies have examined the potential mediating roles of anxiety and depression in the relationship between PE and LS/QOL among Chinese adolescents during the pandemic. Understanding these pathways is essential for developing targeted interventions.

Building on these gaps and grounded in the biopsychosocial framework, this study specifically proposes that anxiety and depressive symptoms serve as critical psychological mediators. We investigate PE’s dual pathways in a sample of Chinese adolescents seeking psychological services: directly enhancing LS and QOL, and indirectly buffering mental health risks through the alleviation of these core emotional symptoms. By focusing on this high-risk group within China’s unique pandemic and academic context, we aim to clarify the mechanisms through which PE supports well-being. To our knowledge, this is the first study to bridge PE’s mental health benefits with its role in fostering LS and QOL by explicitly testing the mediating roles of anxiety and depression in this specific population during COVID-19. Our findings aim to guide school-based interventions that integrate PE programs with strategies to address both academic resilience and post-pandemic mental health recovery.

## Methods

### Participants

This cross-sectional study recruited adolescents aged 12 to 17 years who sought psychological services at the Psychology Department of Shandong Provincial Hospital between June 2021 and May 2022, using a consecutive sampling approach. All eligible adolescents visiting the department during this period were sequentially approached and invited to participate. Inclusion criteria were: (1) age 12–17 years; (2) seeking psychological services at the hospital; (3) no physical illness in the preceding 3 months; (4) no online teaching during the previous 3 months due to the COVID-19 pandemic; and (5) written informed consent from both adolescents and guardians (22, 23). Of the 592 initially recruited participants, 392 were included in the final analysis. Exclusions were based on the following: age <12 years (*n* = 4), age >18 years (*n* = 43), a score of ≥13 on the 32-item Hypomania Checklist (*n* = 113), a score of ≥6 on the Mood Disorder Questionnaire (*n* = 25), belonging to an ethnic minority group (*n* = 6), being an elementary school student (*n* = 6), or missing key variables (*n* = 3).

The study protocol was approved by the Ethics Committee of Shandong Provincial Hospital affiliated with Shandong First Medical University (Approval No: SWYX: NO2021-312) and conducted in accordance with the Declaration of Helsinki. Prior to enrollment, participants and their legal guardians received a detailed explanation of the study objectives, procedures, risks, and benefits. Written informed consent was obtained from all parties after confirming their full understanding of the study and their right to withdraw at any time.

### Measures

#### Sociodemographic information

Demographic and health-related information was systematically collected using a standardized data collection sheet completed by the adolescents. The collected variables included age, gender, educational level (categorized as junior or senior high school), sibling status (only child or not), residential setting (rural or urban), perceived parental marital quality (rated as poor, fair, or good), general health status (self-reported as poor, fair, or good), engagement in regular exercise (yes or no), adherence to a regular diet (yes or no), history of mental illness (yes or no), and history of somatic disease (yes or no).

#### Assessment of depressive and anxious symptoms

Depressive and anxiety symptoms among adolescents were assessed using the Patient Health Questionnaire-9 (PHQ-9) (24) and the Generalized Anxiety Disorder-7 (GAD-7) (25), respectively. The PHQ-9 comprises nine items, with total scores ranging from 0 to 27, whereas the GAD-7 consists of seven items and yields total scores from 0 to 21. For both instruments, each item is rated on a four-point Likert scale reflecting symptom frequency over the preceding 2 weeks: 0 = “not at all,” 1 = “several days,” 2 = “more than half the days,” and 3 = “nearly every day.” Higher total scores indicate greater severity of depressive or anxiety symptoms. Symptom severity was categorized based on established cut-off scores. For the PHQ-9, scores of 5, 10, 15, and 20 correspond to mild, moderate, moderately severe, and severe depression, respectively. Similarly, GAD-7 scores of 5, 10, and 15 represent mild, moderate, and severe anxiety, respectively.

#### Quality of life assessment

Quality of life (QOL) was assessed using the 6-item QOL questionnaire (QOL-6) (26). The instrument was adapted for adolescents by modifying the original “work” dimension to “school performance,” an approach previously validated in youth mental health research (27). Participants were asked to rate six life domains—physical health, psychological health, economic circumstances, school performance, family relationships, and non-family social relationships—during the month preceding enrollment, using a 5-point Likert scale from 1 (very poor) to 5 (excellent).

#### Life satisfaction assessment

Life satisfaction (LS) was measured with the Adolescent Students’ Life Satisfaction Scale (ASLSS) (28), which was constructed with reference to the Multidimensional Student Life Satisfaction Scale (MSLSS) (29). The ASLSS comprises 36 items across six domains: family, friendships, environment, school, academic performance, and freedom. Each item is rated on a 7-point Likert scale ranging from 1 (not at all) to 7 (fully). The scale demonstrated good psychometric properties, with a Cronbach’s alpha coefficient of 0.7942 and a split-half reliability of 0.7710.

### Statistical analysis

Statistical analyses were performed using SPSS version 22.0 (IBM Corp., Armonk, NY, United States). A two-tailed *p*-value of less than 0.05 was considered statistically significant. Categorical variables are presented as frequencies and percentages, and continuous variables as mean ± standard deviation (SD). Differences between adolescents with and without regular physical exercise (PE) in continuous measures—including age, GAD-7 score, PHQ-9 score, QOL-6 score, and ASLSS score—were examined using one-way analysis of variance (ANOVA). Differences in categorical variables between groups were assessed using the chi-square test. To explore the potential mediating effects of anxiety, depression, and PE on QOL and life satisfaction (LS), mediation analyses were conducted using the PROCESS macro (version 2.16.3, Model 4) developed by Hayes.^1^ Bootstrapping with 5,000 resamples was used to test the significance of indirect effects, with mediation considered significant if the 95% bias-corrected confidence interval (CI) did not include zero. Additionally, multiple stepwise regression analysis was performed to identify factors associated with QOL and LS.

## Results

### Sociodemographic and psychological scale assessment of adolescents with and without PE

The study included 133(33.93%) regular physical exercisers (PEs) and 259(66.07%) non-regular physical exercisers (NPEs). As shown in Table 1, sociodemographic characteristics were compared between the two groups. One-way ANOVA revealed no significant age difference between groups (*p* > 0.05). Chi-square analyses further indicated no statistically significant differences in gender, sibling status, parental marital quality, history of somatic disease, or mental illness (all *p* > 0.05). However, significant differences emerged in educational level, residence, general health status, and regular dietary habits (all *p* < 0.05).

**Table 1:** Sociodemographic of regular physical exercisers (PEs) and non-regular physical exercisers (NPEs).

Variables		NPEs (*n* = 259)	PEs (*n* = 133)	*F*/χ^2^	*p*
		mean ± sd.	mean ± sd.		
Age (years)		15.19 ± 1.43	14.93 ± 1.55	2.679	0.103
		n (%)	n (%)		
Gender	Female	166(67.48)	80(32.52)	0.584	0.445
	Male	93(63.70)	53(36.30)		
Education level	Junior high school	92(58.97)	64(41.03)	5.822	0.016^*^
	Senior high school	167(70.76)	69(29.24)		
Siblings	Single	71(60.68)	46(39.32)	2.160	0.142
	Non-Single	188(68.36)	87(31.64)		
Residence	Rural	82(75.93)	26(24.07)	6.458	0.011^*^
	Urban	177(62.32)	107(37.68)		
Quality of parents’ marriages	Poor	42(72.41)	16(27.59)	2.728	0.256
	Fair	106(68.39)	49(31.61)		
	Good	111(62.01)	68(37.99)		
General health condition	Poor	48(85.71)	8(14.29)	34.408	0.000^**^
	Fair	178(69.80)	77(30.20)		
	Good	33(40.74)	48(59.26)		
Regular diet	No	185(74.30)	64(25.70)	20.603	0.000^**^
	Yes	74(51.75)	69(48.25)		
History of somatic disease	No	230(64.97)	124(35.03)	1.970	0.160
	Yes	29(76.32)	9(23.68)		
History of mental illness	No	237(65.83)	123(34.17)	0.112	0.738
	Yes	22(68.75)	10(31.25)		

PEs, regular physical exercisers; NPEs, non-regular physical exercisers; *p < 0.05; **p < 0.01.

To assess psychological differences, one-way ANOVA was conducted on GAD-7, PHQ-9, QOL-6, and six ASLSS dimensions (family, friendships, environment, school, academic performance, and freedom). As summarized in Table 2, adolescents without PE exhibited significantly higher anxiety (GAD-7: PE = 11.21 vs. non-PE = 13.59; *F* = 19.295, *p* < 0.01) and depression scores (PHQ-9: *F* = 34.987, *p* < 0.01). Conversely, the PE group demonstrated superior QOL-6 scores (*F* = 58.295, *p* < 0.01) and significantly higher satisfaction across all ASLSS dimensions (family, friendships, environment, school, academic performance, and freedom; all *p* < 0.05).

**Table 2:** Psychological scale assessment results of regular physical exercisers (PEs) and non-regular physical exercisers (NPEs).

Variables	NPEs (*n* = 259) (mean ± sd.)	PEs (*n* = 133) (mean ± sd.)	*F*	*p*
GAD-7	13.59 ± 4.93	11.21 ± 5.37	19.295	0.000**
PHQ-9	18.02 ± 5.81	14.14 ± 6.73	34.987	0.000**
Six-Item Quality of Life Questionnaire	15.22 ± 3.43	18.09 ± 3.68	58.295	0.000**
Scale of Adolescent Students’ Life Satisfaction				
Friendship	3.56 ± 1.22	4.37 ± 1.22	38.774	0.000**
Family	3.78 ± 1.53	4.45 ± 1.56	16.615	0.000**
School	2.50 ± 1.37	3.34 ± 1.53	30.300	0.000**
Academics	2.32 ± 1.07	3.33 ± 1.33	66.154	0.000**
Freedom	3.30 ± 1.29	4.08 ± 1.30	31.702	0.000**
Environment	4.06 ± 1.21	4.64 ± 1.13	21.502	0.000**

PEs, regular physical exercisers; NPEs, non-regular physical exercisers; *p < 0.05; **p < 0.01.

### Results of the mediation analysis of PE (X) on QOL-6/ASLSS (Y) through depressive/anxious symptoms

Table 3 presents the comprehensive analysis of the total, direct, and indirect effects of PE on QOL-6, with depressive and anxious symptoms serving as mediators. The total effect of exercise on QOL, mediated by these psychological factors, is substantial, demonstrating that PE significantly enhances QOL. The direct effect of PE—excluding the influence of depressive and anxious symptoms—contributes 50.845 and 68.725% to the improvement in QOL-6. Meanwhile, the indirect effect, facilitated through the alleviation of depressive and anxious symptoms, accounts for 49.155 and 31.275% of the enhancement.

**Table 3:** Total, direct, and indirect effects of regular physical exercise (X) on quality of life (Y) through depressive/anxious symptoms (M) (*N* = 392).

Variables	Effect	Total effect percentage (%)	Estimate	95% CI		Boot SE	*t*	*p*
				Lower	Upper			
PE→GAD-7→QOL-6	Indirect effect	31.275	0.896	0.060	0.165	0.027	33.218	0.000**
PE→QOL-6	Direct effect	68.725	1.970	1.336	2.603	0.323	6.095	0.000**
PE→QOL-6	Total effect	100	2.866	2.130	3.602	0.375	7.635	0.000**
PE→PHQ-9→QOL-6	Indirect effect	49.155	1.409	0.114	0.237	0.032	44.376	0.000**
PE→QOL-6	Direct effect	50.845	1.457	0.863	2.052	0.303	4.805	0.000**
PE→QOL-6	Total effect	100	2.866	2.130	3.602	0.375	7.635	0.000**

PE, physical exercise; GAD-7, Generalized Anxiety Disorder-7; QOL-6, 6-items Quality of Life questionnaire; PHQ-9, the Patient Health Questionnaire-9. **p < 0.01.

Table 4 details the total, direct, and indirect effects of PE on LS domains, with depressive symptoms as the mediator. The total effects across all domains were significant. A key finding is that the direct effect of PE on family satisfaction was precisely zero (0%). For other domains, the direct effects of PE (independent of depressive symptoms) contributed 65.217% (friendships), 51.875% (school), 69.933% (academics), 45.567% (freedom), and 39.934% (environment) to the respective satisfaction improvements. Correspondingly, the indirect effects mediated through depressive symptom reduction accounted for 34.783, 48.125, 30.067, 54.433, and 60.066% of the effects in those same domains, and for 100% of the effect on family satisfaction.

**Table 4:** Total, direct, and indirect effects of regular physical exercise (X) on perceived life satisfaction (Y) through depressive symptoms (M) (*N* = 392).

Variables	Effect	Total effect percentage (%)	Estimate	Boot 95% CI		Boot SE	t	*p*
				Lower	Upper			
PE→PHQ-9→ASLSS friendship	Indirect effect	34.783	0.281	0.061	0.153	0.024	11.818	0.000**
PE→ASLSS friendship	Direct effect	65.217	0.527	0.280	0.774	0.126	4.175	0.000**
PE→ASLSS friendship	Total effect	100	0.808	0.554	1.062	0.130	6.227	0.000**
PE→PHQ-9→ASLSS family	Indirect effect	100	0.384	0.070	0.164	0.024	16.021	0.000**
PE→ASLSS family	Direct effect	0	0.287	−0.023	0.597	0.158	1.817	0.070
PE→ASLSS family	Total effect	100	0.671	0.348	0.993	0.165	4.076	0.000**
PE→PHQ-9→ASLSS school	Indirect effect	48.125	0.404	0.078	0.182	0.027	15.053	0.000
PE→ASLSS school	Direct effect	51.875	0.435	0.156	0.715	0.142	3.055	0.002**
PE→ASLSS school	Total effect	100	0.839	0.540	1.138	0.152	5.505	0.000**
PE→PHQ-9→ASLSS academic performance	Indirect effect	30.067	0.304	0.068	0.163	0.024	12.525	0.000**
PE→ASLSS academic performance	Direct effect	69.933	0.707	0.475	0.939	0.118	5.977	0.000**
PE→ASLSS academic performance	Total effect	100	1.011	0.767	1.255	0.124	8.134	0.000**
PE→PHQ-9→ASLSS freedom	Indirect effect	54.433	0.423	0.093	0.205	0.029	14.761	0.000**
PE→ASLSS freedom	Direct effect	45.567	0.354	0.112	0.597	0.124	2.870	0.004**
PE→ASLSS freedom	Total effect	100	0.778	0.507	1.049	0.138	5.630	0.000**
PE→PHQ-9→ASLSS environment	Indirect effect	60.066	0.352	0.086	0.188	0.026	13.545	0.000**
PE→ASLSS environment	Direct effect	39.934	0.234	0.006	0.462	0.116	2.008	0.045*
PE→ASLSS environment	Total effect	100	0.586	0.338	0.833	0.126	4.637	0.000**

PE, physical exercise; ASLSS, Adolescent Students’ Life Satisfaction Scale; PHQ-9, the Patient Health Questionnaire-9. *p < 0.05; **p < 0.01.

Table 5 offers the total, direct, and indirect effects of PE on adolescents’ LS, with anxious symptoms serving as mediators. The analysis reveals a significant overall influence of PE on satisfaction in areas such as friendships, family, school, academics, freedom, and environment, underscoring the substantial enhancement of LS through PE. The direct effect of PE—unaffected by anxious symptoms—contributes 77.377, 73.602, 73.203, 83.503, 68.182, and 67.112% to satisfaction improvements in these respective areas. Conversely, the indirect effect, mediated through the alleviation of anxious symptoms, accounts for the remaining 22.623, 26.398, 26.797, 16.497, 31.818, and 32.888% of the satisfaction enhancements in those areas.

**Table 5:** Total, direct, and indirect effects of regular physical exercise (X) on perceived life satisfaction (Y) through anxious symptoms (M) (*N* = 392).

Variables	Effect	Total effect percentage (%)	Estimate	Boot 95% CI		Boot SE	t	*p*
				Lower	Upper			
PE→GAD-7→ASLSS friendship	Indirect effect	22.623	0.183	0.032	0.109	0.020	9.359	0.000**
PE→ASLSS friendship	Direct effect	77.377	0.625	0.378	0.872	0.126	4.959	0.000**
PE→ASLSS friendship	Total effect	100	0.808	0.554	1.062	0.130	6.227	0.000**
PE→GAD-7→ASLSS family	Indirect effect	26.398	0.177	0.024	0.090	0.017	10.526	0.000**
PE→ASLSS family	Direct effect	73.602	0.494	0.173	0.814	0.164	3.017	0.003**
PE→ASLSS family	Total effect	100	0.671	0.348	0.993	0.165	4.076	0.000**
PE→GAD-7→ASLSS school	Indirect effect	26.797	0.225	0.034	0.115	0.021	10.948	0.000**
PE→ASLSS school	Direct effect	73.203	0.614	0.326	0.903	0.147	4.170	0.000**
PE→ASLSS school	Total effect	100	0.839	0.540	1.138	0.152	5.505	0.000**
PE→GAD-7→ASLSS academic performance	Indirect effect	16.497	0.167	0.030	0.101	0.018	9.341	0.000**
PE→ASLSS academic performance	Direct effect	83.503	0.844	0.606	1.082	0.121	6.954	0.000**
PE→ASLSS academic performance	Total effect	100	1.011	0.767	1.255	0.124	8.134	0.000**
PE→GAD-7→ASLSS freedom	Indirect effect	31.818	0.248	0.044	0.132	0.022	11.098	0.000**
PE→ASLSS freedom	Direct effect	68.182	0.530	0.277	0.784	0.129	4.099	0.000**
PE→ASLSS freedom	Total effect	100	0.778	0.507	1.049	0.138	5.630	0.000**
PE→GAD-7→ASLSS environment	Indirect effect	32.888	0.193	0.037	0.117	0.020	9.515	0.000**
PE→ASLSS environment	Direct effect	67.112	0.393	0.155	0.631	0.122	3.235	0.001**
PE→ASLSS environment	Total effect	100	0.586	0.338	0.833	0.126	4.637	0.000**

PE, physical exercise; ASLSS, Adolescent Students’ Life Satisfaction Scale; GAD-7, Generalized Anxiety Disorder-7; *p < 0.05; **p < 0.01.

### Results of stepwise linear regression analyses on adolescents’ QOL-6 and ASLSS dimensions

In a stepwise regression analysis, we examined gender, age, educational level, sibling status, residence, parental marital quality, general health status, PE, regular diet, history of somatic disease, history of mental illness, GAD-7, and PHQ-9 as independent variables, with QOL-6 and ASLSS six dimensions (family, friendships, environment, school, academic performance, and freedom) as dependent variables. The model automatically identified that the regression coefficient for PE was 0.917 (*t* = 3.266, *p* = 0.001 < 0.01), indicating a significant positive impact on QOL-6(see Supplementary Table 1). The regression coefficient for PE was 0.457 (*t* = 3.584, *p* = 0.000 < 0.01) for LS with friendship, suggesting a significant positive effect (see Supplementary Table 2). For LS with school, the coefficient was 0.331 (*t* = 2.300, *p* = 0.022 < 0.05), indicating a notable positive influence (see Supplementary Table 4). In terms of satisfaction with academic performance, PE showed a coefficient of 0.707 (*t* = 5.977, *p* = 0.000 < 0.01), highlighting a significant positive relationship (see Supplementary Table 5). Furthermore, the regression coefficient for freedom satisfaction was 0.335 (*t* = 2.747, *p* = 0.006 < 0.01), further underscoring a significant positive effect of PE (see Supplementary Table 6). In relation to satisfaction with family and environment, PE was not included in the regression equation, indicating that PE did not exert a significant effect on these dimensions (refer to Supplementary Tables 3, 7).

## Discussion

The analysis in this study revealed no significant demographic differences in age, gender, or family background between adolescents who engaged in regular physical exercise and those who did not. However, findings indicated that adolescents who exercised regularly demonstrated multiple advantages in health and subjective well-being compared to their less active peers. These advantages included better general health and dietary habits, as well as significantly lower levels of anxiety and depression. Regular exercisers also reported a markedly higher QOL and greater satisfaction across multiple domains, including friendships, family interactions, school experience, academic performance, autonomy, and living environment. These findings suggest multifaceted benefits associated with physical exercise among adolescents, particularly in mitigating symptoms of anxiety and depression, enhancing LS, and improving overall QOL. These results are consistent with existing literature emphasizing the positive role of physical activity in promoting psychological and emotional well-being across diverse populations (30–35).

Previous studies have shown that regular exercise can reduce the probability of developing anxiety and depressive disorders (30), and that higher levels of habitual physical activity are associated with higher scores on measures of positive affect, including interest, excitement, enthusiasm, and alertness (36). Potential underlying mechanisms may involve improvements in neuroendocrine function, increased endorphin release, and enhanced self-efficacy (37). Research indicates that regular physical activity can strengthen hypothalamic–pituitary–adrenal axis function, improve mood and quality of life, promote better sleep, and alleviate symptoms of various mental disorders (38). Furthermore, participation in physical activities provides adolescents with a sense of achievement and social belonging, which further contributes to improved mental health and life satisfaction (31).

While numerous studies have explored the associations between physical exercise and mental health, research on the specific mechanisms underlying these relationships, particularly pathways elucidated through mediation analysis, remains relatively underexplored in the existing literature. In this study, conducted within the context of the COVID-19 pandemic, we employed mediation analysis to examine the influence of physical exercise on QOL and LS. The results demonstrated that regular physical exercise was associated with improved QOL and LS through both direct and indirect pathways, the latter specifically involving the alleviation of anxiety and depressive symptoms. This pathway suggests that interventions targeting regular physical exercise in adolescents may yield significant benefits for psychological outcomes and overall well-being.

Regression analyses further supported that, even after controlling for psychological distress, regular physical exercise was associated with increased life satisfaction in the domains of friendships, school, academic performance, and autonomy. This direct effect may stem from the improved physical health, increased energy levels, and enhanced opportunities for social interaction afforded by participation in physical activities (39). A notable distinction emerged in the pathways through which physical exercise related to different life domains. As detailed in Table 4, its positive association with family satisfaction was entirely indirect, fully mediated by the reduction of depressive symptoms (i.e., 100% mediation). This pattern suggests that any benefit of exercise for perceived family relationships operates primarily through its role in improving the adolescent’s own emotional state. In contrast, for domains such as friendships, school, and environment, physical exercise demonstrated both substantial direct effects and significant indirect effects via symptom alleviation (partial mediation). These findings indicate that the influence of physical exercise is domain-specific. The lack of a direct effect on family satisfaction likely reflects that this domain is influenced more by external, relational factors (e.g., family dynamics, communication patterns, parental support) that are not directly altered by individual exercise behavior. Conversely, domains like school performance or autonomy may be more directly impacted by the cognitive, energetic, and self-efficacy benefits of exercise. Moreover, involvement in sports or group activities helps foster social connections and a sense of community belonging, which are key components of life satisfaction, particularly in friendship and school domains. This study also highlights other factors influencing adolescents’ QOL and LS, such as dietary habits, parental marital quality, and personal health status. These findings echo broader research indicating that lifestyle factors play a crucial role in shaping the overall well-being of adolescents (40, 41). Future research should consider these multifactorial influences and explore how they interact with physical exercise to affect adolescents’ mental health and life satisfaction.

This study has several limitations. First, the cross-sectional design and reliance on self-reported data from adolescents to assess variables such as QOL, LS, physical exercise, and symptoms of anxiety and depression may introduce reporting biases. Second, due to the methodological nature of mediation analysis, the mediation analyses in this study were based on cross-sectional correlational data, which presents challenges in definitively establishing causality. Consequently, inherent uncertainty exists in the interpretation of these findings. Third, our data were primarily derived from adolescents seeking mental health services at a single center, which may limit the generalizability of the results to the broader adolescent population. Additionally, variations in cultural and environmental contexts could influence lifestyle, emotional states, QOL, and LS, further constraining the generalizability of the findings. Finally, the study did not control for several potential confounding factors, such as socioeconomic status, parental education level, screen time, and sleep quality, which may influence the relationship between physical activity and mental health outcomes. Future research should aim to include larger sample sizes and participants from diverse cultural and socioeconomic backgrounds to provide more generalizable results and enhance understanding of how these variables interact with physical exercise and mental health outcomes.

## Conclusion

This study identified differences in socio-demographic characteristics, emotional states, and levels of subjective well-being between adolescents who regularly engaged in physical exercise and those who did not. Further mediation analysis revealed that physical exercise was associated with quality of life and life satisfaction through both direct and indirect pathways. These findings support the integration of physical exercise into health education policies as a strategy to address mental health challenges among adolescents, particularly in the current context where Chinese adolescents face significant pressures across multiple domains. While acknowledging limitations related to sample diversity and potential confounders, this study establishes a foundation for future research. Expanding investigations to more diverse populations and conducting cross-cultural comparisons will be crucial for developing universally effective strategies. Ultimately, promoting an active lifestyle is essential for supporting adolescent mental health and building psychological resilience.

## Data Availability

The data analyzed in this study is subject to the following licenses/restrictions: the datasets generated and analyzed in this study are available from the corresponding author, Ping Sun and Guoqing Zhao, upon reasonable request. Requests to access these datasets should be directed to Guoqing Zhao, zhaoguoqing0917@163.com and Ping Sun, qdsunping99@sina.com.

## Footnotes

1
http://www.afhayes.com
